# Cervical Hyperextension Causes Acute Cerebral Congestion in Non-Anesthetized Healthy Adults: An Observational Self-Controlled Design Study

**DOI:** 10.3390/medicina61101791

**Published:** 2025-10-03

**Authors:** Ozlem Ersoy Karka, Derya Guclu, Ilknur S. Yorulmaz, Mehmet A. Sungur, Gizem Demir Senoglu, Gulbin Sezen, Yavuz Demiraran

**Affiliations:** 1 Department of Anesthesiology and Reanimation, Faculty of Medicine, Duzce University, Duzce 81010, Turkey; issekerdtf@gmail.com (I.S.Y.); gizem123demir@hotmail.com (G.D.S.); gysezen@hotmail.com (G.S.); demiraran@gmail.com (Y.D.); 2 Department of Medical Imaging and Radiodiagnostics, Faculty of Medicine, Duzce University, Duzce 81010, Turkey; deryasr@hotmail.com; 3 Department of Medical Informatics and Biostatistics, Faculty of Medicine, Duzce University, Duzce 81010, Turkey; malisungur@yahoo.com

**Keywords:** neck, head movements, healthy volunteers, ultrasonography, Doppler, neurophysiological monitoring, optic nerve, cognitive tests, vasogenic cerebral edema

## Abstract

*Background and Objectives*: Severe complications associated with cervical hyperextension during general anesthesia have been reported. The question is whether some of the cerebral/spinal ischemic complications could be partially related to the position itself. Cerebral oximetric monitoring, combined with optic nerve sheath diameter and cognitive function in non-anesthetized healthy volunteers, should provide more extensive information about the effects of cervical hyperextension, independent of anesthesia. *Materials and Methods*: 51 healthy volunteers with no vertebrobasilar abnormalities completed the study and were included in statistical analysis. Primary outcomes were cervical blood flow and cerebral relative hemoglobin change. The secondary outcomes were optic nerve sheath diameter and cognitive function assessment. After baseline Doppler ultrasonography of the cervical vessels, Mini-Mental State Examination, Montreal Cognitive Assessment, and optic nerve sheath diameter measurements at T0, volunteers underwent cervical hyperextension > 30°, with assessments repeated at the first and 30th minutes (T1, T30). Relative total, oxi-, and deoxyhemoglobin changes were assessed every 5 min. *Results*: Peak systolic velocities in the jugular veins at T1 and T30 were significantly lower than those at baseline (p1 and p2 < 0.001). After hyperextension, deoxyhemoglobin changes significantly increased at T1 and T30 (p1 < 0.001). The optic nerve sheath diameter increased at T30 compared to the baseline (*p* < 0.001). Cognitive scores improved at T30 (*p* = 0.044 and *p* < 0.001, respectively). *Conclusions*: Cervical hyperextension causes a significant increase in relative total and deoxyhemoglobin changes, which are related to acute cerebral congestion by severely impairing cerebral venous flow. A significant change in optic nerve sheath diameter indirectly indicates increased intracranial pressure.

## 1. Introduction

Cervical hyperextension is frequently employed in operating rooms during many surgical procedures including cardiovascular, head/neck reconstruction, dental/maxillary surgeries, tonsillectomy, thyroidectomy, parathyroidectomy, cervical disc herniation repair. Several complications associated with these surgeries have been reported in the literature. These include postoperative pain, changes in blood flow velocity or injury to the cervical vessels, spinal cord damage, central cord syndrome, stroke, quadriplegia, damage to the thyroid cartilage and larynx, bradycardia, and asystole [1,2,3,4,5,6,7,8,9,10,11,12,13,14,15]. Although the consequences of surgeries in cervical hyperextension have been well known for a long time, it is still impossible to avoid.

Current research on the clinical effects of cervical hyperextension involves measuring cerebral/cervical hemodynamic changes using Doppler ultrasound (DUS), magnetic resonance angiography (MRA), and cerebral oximetry assessing oxygen saturation (SO) and hemoglobin (Hb) levels in brain tissue through near-infrared spectroscopy [5,16,17,18,19,20,21,22,23]. However, in studies conducted on surgical populations under general anesthesia, it is possible that the effects of general anesthesia partially influenced the results [5,19]. This raises the question of whether some of the aforementioned cerebral or spinal ischemic complications could be partially related to the position itself. Nevertheless, multifactorial monitoring, namely cerebral oximetry combined with optic nerve sheath diameter (ONSD) and cognitive function, can provide more extensive information about the effects of cervical hyperextension. Studies on the effects of cervical hyperextension in awake individuals are limited. Consequently, it is difficult to discern the proportion of neurological complications during surgery in this position, which can be attributed to the position itself, as opposed to those caused by surgery or anesthesia.

Several previous studies have investigated the hemodynamic effects of cervical hyperextension, mostly in surgical populations under general anesthesia. For example, Fudickar et al. demonstrated a significant (>20%) reduction in middle cerebral artery blood flow with head and neck rotation and extension in anesthetized patients [22]. Saracoğlu et al. reported decreased common carotid artery blood flow during thyroidectomy procedures performed under general anesthesia, while cerebral oxygen saturation was preserved, likely due to the reduced cerebral metabolic rate caused by anesthesia [5]. Similarly, Lim et al. showed increased optic nerve sheath diameter during robot-assisted thyroidectomy, which they attributed to impaired jugular venous drainage [24]. However, the confounding effects of anesthesia, surgical stress, and neuromuscular blockade in these studies limit their ability to isolate the pure impact of cervical positioning.

To our knowledge, no previous study has directly examined the isolated effects of cervical hyperextension in non-anesthetized healthy adults. The present study was therefore designed to fill this gap.

This study aimed to investigate the effects of cervical hyperextension on cerebral hemodynamics and cognitive function in non-anesthetized healthy adults. The primary outcomes were changes in cervical blood flow and cerebral oximetry, while secondary outcomes were changes in ONSD and cognitive function. We hypothesized that remaining in a cervical hyperextension position in non-anesthetized healthy adults would decrease cervical blood flow velocities, cerebral oxygen saturation, and cognitive scores, without the confounding effects of anesthesia or comorbidities.

## 2. Materials and Methods

Ethics approval for this study (number: 2020/03, date: 3 February 2020) was provided by Duzce University Non-Invasive Health Research Ethics Committee, Duzce, Turkiye (Chairperson Prof G. Sezen). This study was registered in the clinicaltrials.gov database (NCT05576857) and financially supported by the Duzce University Scientific Projects Coordination Office (DUBAP, Duzce, Turkiye) with Project Number 2021.04.02.1199. This study was conducted in accordance with the Declaration of Helsinki at the Duzce University Health Research Center between 2021–2024.

**Figure 1:**
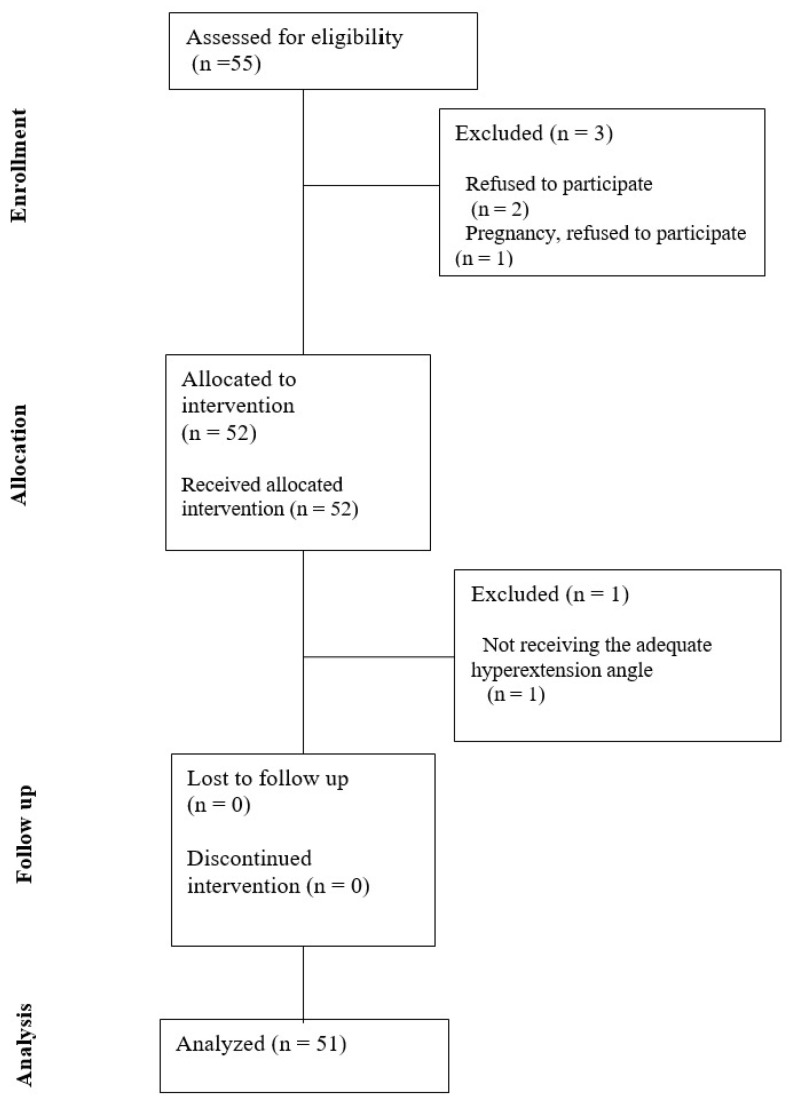
Flow diagram of volunteer recruitment.

This study was designed as a prospective, observational, self-controlled study conducted on non-anesthetized healthy adults, specifically aiming to isolate the effects of cervical hyperextension on cerebral hemodynamics by eliminating potential confounding influences of anesthesia and comorbidities, with each participant serving as their own control in the absence of a separate control group. Fifty-five healthy volunteers aged 18–65 who signed a written informed consent were included (Figure 1). All volunteers (n = 55) underwent noncontrast cervical MRA showing vertebrobasilar circulation. None of the subjects had abnormalities in vertebrobasilar circulation, which was an exclusion criterion. Further exclusion criteria were systemic diseases, cardiac diseases, neurological diseases, hemoglobinopathies, and anemia.

Each volunteer was contacted again and at an individual appointment the Mini-Mental State Examination (MMSE) and Montreal Cognitive Assessment (MoCA) were administered [25,26]. On the same day, the volunteer was placed supine on a stretcher within the Radiology Unit and monitored using a Root O3™ Regional Oximetry device (Masimo Corporation, Irvine, CA, USA). Two electrodes were placed on bilateral foreheads to track cerebral SO and relative changes in total (ΔcHbi), deoxygenated (ΔHHbi), and oxygenated (ΔO2Hbi) hemoglobin. Thereafter, cervical blood flow measurements were performed bilaterally with DUS while the subject was supine and breathing spontaneously in room air. Acuson-S2000^®^ (Siemens AG Healthcare, Erlangen, Germany) device with a 9L4 (4–9 MHz) linear probe was used for DUS. Bilateral diameter (Diam), peak systolic velocity (PSV), end-diastolic velocity (ED), resistive index (RI), blood flow volume (BFlow) baseline values of arteria carotis communis (CCA), arteria carotis interna (ICA) and vertebral artery (VA), and Diam and PSV baseline values of vena jugularis interna (VJI) and ONSD were measured at baseline (T0). ONSD was measured three times 3 mm behind the point where the optic nerve connects to the eyeball, and the mean of three measurements was recorded. After baseline measurements, the neck of each volunteer was maximally hyperextended at an angle > 30°, in which the volunteer did not experience neck discomfort or pain. A standard under-shoulder elevator pillow was used, and the head-neck region was supported with soft gel pads. The hyperextension angle was measured using a goniometer (Yildizlar^®^ Istanbul, Turkey) with pivot point on tuberculum majus on the shoulder, fixed arm parallel to the ground, and movable arm parallel to the vertical axis of the head. Immediately after positioning, cerebral oximeter and cervical blood flow measurements were repeated (T1). Volunteers (n = 51) were kept in cervical hyperextension for 30 min, and cerebral oximetry and Hb levels were recorded every 5 min. At the 30th minute, cervical blood flow and ONSD measurements, MMSE, and MoCA were readministered (T30).

The 30 min duration was selected based on previous findings indicating the onset of measurable hemodynamic changes within this timeframe [21]. The same experienced radiologist performed MRA and DUS assessments. Positioning, measurement of hyperextension angle, monitoring parameters, and cognitive examinations were performed and recorded by the same experienced anesthesiologist. Variables measured at T0, T1, and T30 and between T1 and T30 are shown in Supplementary Materials Table S1.

### Statistical Analysis

Power analysis considered the change in CCA blood flow velocity in the study by Saraçoğlu et al. [5]. This analysis was performed using G*Power v.3.1.9 (Heinrich Heine University, Dusseldorf, Germany). This study aimed to assess the moderate correlation between the measurements and the statistical significance of a 65-unit difference (effect size 0.412). To achieve 80% power and 5% type I error, a minimum of 49 patients were required. We included a dropout rate of 10% in our projections.

IBM SPSS Statistics for Windows, Version 22.0 (IBM Corp., Armonk, NY, USA) was used for statistical data evaluation. The normality assumption was examined using the Shapiro–Wilk test. Descriptive statistics were presented as means and standard deviations. Paired sample *t*-test was used to compare two measurements over different periods. Comparison of variables measured in three or more different periods was performed with repeated measures of analysis of variance, and a post hoc LSD test was used when needed. Repeated measures analysis of covariance was also used to control for the potential confounding effects of the independent variables. Pearson correlation coefficient was used to analyze the correlation between quantitative variables. Differences were considered statistically significant at a *p* value of 0.05.

## 3. Results

This study included 55 participants. During the study, one participant was excluded due to pregnancy, two discontinued participation, and one was excluded for failing to achieve a cervical hyperextension angle > 30° (Figure 1). Demographic and clinical characteristics of the 51 participants who completed the study are presented in Table 1. The hyperextension angle was between 30–52° with a mean ± SD of 45.02° ± 5.09°.

### 3.1. Cervical Blood Flow Measurements

Table 2 shows the DUS measurements with p_1_ representing T1 vs. T0, p_2_: T30 vs. T0, and p_3_: T30 vs. T1. When the right CCA was evaluated in terms of Diam and PSV, it was shown that at T1 and T30, Diam decreased (p_1_ = 0.019, p_2_ = 0.002) and PSV increased (p_1_ = 0.001, p_2_ = 0.005) significantly compared to the baseline. In the left CCA, Diam was significantly reduced at T1 (p_1_ = 0.003) and PSV increased significantly at T30 compared to baseline (p_2_ = 0.024). Comparing the right and left CCA, ED measurements at T1 and T30 were significantly increased compared to baseline (right CCA: p_1_ = 0.001, p_2_ = 0.004; left CCA: p_1_ < 0.001, p_2_ < 0.001). Regarding the RI, no change was observed in position at any time interval for either CCA. In the comparisons regarding BFlow of the right CCA, measurements at T30 were significantly higher than those at baseline (p_2_ = 0.038). In comparisons of left CCA BFlow, both T1 and T30 measurements were significantly higher than baseline (p_1_ = 0.036, p_2_ = 0.014).

T1 and T30 Diam values of the right ICA decreased significantly compared to baseline (p_1_ = 0.019, p_2_ = 0.015). There was no significant change in the left ICA Diam. When PSV measurements of ICAs were compared to baseline, T1 and T30 values were significantly decreased on both sides (Right ICA: p_1_ = 0.031, p_2_= 0.005. Left ICA: p_1_ = 0.035, p_2_= 0.002). The ED, RI, and BFlow of both ICAs did not change significantly.

Comparing the Diam of bilateral VJIs, no statistically significant difference was found when comparing T1 and T30 measurements with the basal and the value at each period with the previous. When PSV of the right and left VJIs were compared to baseline, the findings showed a significant decrease immediately after the cervical hyperextension position (T1) and were maintained until the end of the position (T30) (Right VJI: p_1_ < 0.001, p_2_ < 0.001. Left VJI: p_1_ < 0.001, p_2_ < 0.001). (Supplementary Materials Figure S1)

In measurements related to VA, no statistically significant finding was observed, except that the PSV of the left VA was significantly decreased at T30 compared to baseline (p_2_ = 0.040).

### 3.2. Cerebral Oximetry Parameters

Table 3, with p_1_ representing compared to basal and p_2_: compared to previous, and Supplementary Materials Figure S2 show a significant decrease in the SO levels immediately after the cervical hyperextension position (T1) (p_1_ < 0.001) and is maintained in this manner at all time periods. When comparing ΔcHbi values with baseline, the values measured at all periods after the hyperextension position were shown to be significantly increased (p_1_ < 0.001, except for Left ΔcHbi-T30: p_1_ = 0.002). When comparing ΔHHbi with baseline, the values measured at all periods after hyperextension were significantly increased (p_1_ < 0.001). When comparing ΔO_2_Hbi with baseline, values measured at the fifth, tenth, and 15th minutes after hyperextension were significantly increased (p_1_ = 0.007, p_1_ = 0.013, p_1_ = 0.032, respectively) (Table 3). Supplementary Materials Figures S3 and S4 illustrate significant increases in ΔcHbi and ΔHHbi immediately following cervical hyperextension, which were subsequently maintained. In contrast, ΔO_2_Hbi exhibited a notable increase up to 15 min; thereafter, it slightly decreased and remained above the baseline.

Values are presented as mean ± SD. p-values reaching statistical significance are presented in bold. p_1_: compared to the basal, p_2_: compared to the previous. SO: cerebral oxygen saturation; ΔcHbi: relative total hemoglobin change; ΔHHbi: relative deoxyhemoglobin change; ΔO_2_Hbi: relative oxyhemoglobin change. basal: basal measurements made at T0 (T0: Time frame before hyperextension when the basal measurements were made.); T1: measurements made at the first minute after hyperextension; T5: measurements made at the 5th minute after hyperextension; T10: measurements made at the 10th minute after hyperextension; T15:measurements made at the 15th minute after hyperextension; T20: measurements made at the 20th minute after hyperextension; T25: measurements made at the 25th minute after hyperextension; T30: measurements made at T30 (T30: 30th minute after hyperextension). η^2^: partial eta squared, F: repeated measures ANOVA.

### 3.3. Cognitive Function Examinations

MMSE and MoCA scores were significantly higher at T30 than at baseline (MMSE, *p* = 0.044; MoCA, *p* < 0.001) (Table 1).

### 3.4. Optic Nerve Sheath Diameter Measurements

**Figure 2:**
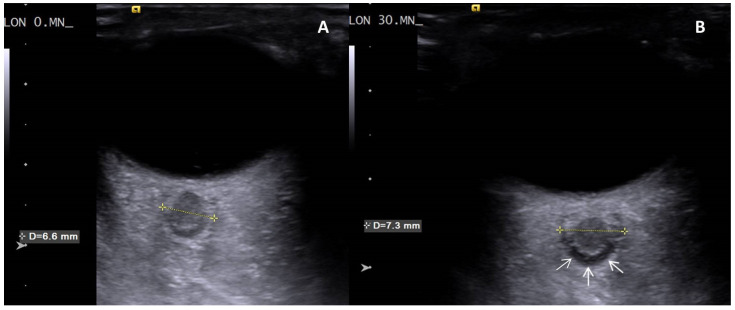
Transorbital ultrasound visualization of the axial plane of the left eye. The optic nerve appears hypoechoic and is surrounded by the hyperechoic optic nerve sheath. (**A**) Optic nerve sheath in a volunteer with normal intracranial pressure at T0 showing no visible subarachnoid space around the optic nerve. (**B**) Optic nerve sheath in the same volunteer at the 30th minute of cervical hyperextension position demonstrates a significant subarachnoid space around the optic nerve (white arrows), indicating elevated intracranial pressure.

A significant increase was found when T30 values were compared to the basal values for the right and left ONSD (*p* < 0.001, *p* < 0.001, respectively) (Table 4, Figure 2, Supplementary Materials Figure S5).

## 4. Discussion

Analysis of DUS findings showed a decrease in Diam and PSV of the ICA and an increase in ED and BFlow of the CCA immediately after cervical hyperextension, which persisted throughout the hyperextension period. PSV in the VJIs decreased significantly, suggesting that blood was pooled in the brain tissue due to inadequate venous drainage. Meanwhile, arterial blood supply was maintained. This was preceded by a significant increase in ΔcHbi and ΔHHbi in both hemispheres, and an increase in ONSD bilaterally.

It seems beneficial to follow cerebral oximeter and interpret the changes that occur compared to baseline. This interpretation allows the evaluation of the underlying cause of cerebral desaturation after hyperextension. In previous studies, changes in Hb spectral absorption have been classified according to three variations in cerebral desaturation. One among three is that ΔO_2_Hbi stays constant, while the values for ΔcHbi and ΔHHbi increase, which are termed as ‘acute cerebral congestion’ [27,28,29,30]. In our study, the ΔO_2_Hbi values changed slightly, whereas the ΔcHbi and ΔHHbi values increased significantly and remained high, consistent with acute cerebral congestion [27].

In addition to changes in cerebral oximetry, the increase in ONSD further supports the finding of acute cerebral congestion. The optic nerve, an extension of the brain, is encased by the meninges and links the cerebrospinal fluid of the optic nerve sheath (ONS) directly with that of the subarachnoid space. It is known that an increase in intracranial pressure (ICP) results in an increase in fluid in the ONS, leading to an increase in ONSD. Ultrasonographic measurement of ONSD allows indirect assessment of ICP increase with sensitivity and specificity of 75–100% and 100%, respectively [31,32,33,34,35,36,37,38,39]. Consequently, while cervical hyperextension had minor impact on cerebral arterial blood flow in our study, it significantly hindered venous drainage, resulting in acute cerebral congestion. Our study’s key findings showed a significant increase in ONSD bilaterally due to elevated ICP in the acute cerebral congestion clinic (Figure 2). In a study by Lim et al., ONSD increased significantly after CO_2_ neck insufflation and decreased after deflation in robot-assisted thyroidectomies. They explained this by addressing the reduction in jugular venous blood flow due to higher pressure in the neck; however, they overlooked the effect of cervical hyperextension. Notably, ONSD remained above baseline until just before PACU discharge, although this was not statistically significant [24]. This finding and discussion are similar to the acute cerebral congestion observed in our study.

Importantly, Weintraub et al. concluded that cervical hyperextension did not cause ischemic symptoms for up to 12 min. If it lasts longer than 12 min, it is an overlooked potential hemodynamic factor that may play a crucial role in the risk of perioperative stroke. Unlike our study, the study population of Weintraub et al. had comorbidities such as diabetes, atherosclerosis, and clinically silent old infarct areas [21]. This fact allowed us to evaluate the effect of the cervical hyperextension position by eliminating other factors, as we aimed in our study.

Köse and Hatipoğlu evaluated the mean cerebral blood flow volume of the middle cerebral artery (MCA) using transcranial DUS in cranial surgery patients by providing various head-neck positions including flexion and extension. They concluded that head flexion and extension applied in addition to 30° head elevation caused a decrease in cerebral blood flow, but did not reach statistical significance [18]. The study sample consisted of patients with high ICP. The results of this study may be explained by the regulation of cerebral blood flow volume owing to the activation of cerebral autoregulation in the high ICP group. Cerebrovascular autoregulation is vital for maintaining cerebral blood flow and perfusion. It is defined as the capacity of cerebral blood vessels to maintain stable cerebral blood flow despite changes in blood pressure [28,40]. In healthy individuals, cardiovascular, respiratory, and neural physiological interactions maintain adequate cerebral blood flow by modulating the hydrodynamic parameters [41]. In another study conducted by Saracoglu et al. CCA average velocity, PSV, Diam, and BFlow measured with DUS and cerebral SO were followed up in patients undergoing thyroidectomy. CCA-BFlow decreased by 35% after cervical extension in thyroidectomies compared to the basal. Cerebral SO increased after induction and persisted until the end of the surgery. No cerebral desaturation occurred. The research indicates that the reduction in CCA-BFlow is probably a result of decreased mean arterial pressure, which is attributed to a decrease in systemic vascular resistance during general anesthesia. They noted that the reason for maintaining cerebral oxygenation is the reduced cerebral metabolic rate and oxygen consumption due to general anesthesia [5]. Unlike ours, this study focused only on the anterior circulation of the brain, did not include measurements of vertebrobasilar circulation, and was unable to eliminate anesthetic effects. Similarly, Fudickar et al. showed by transcranial DUS that head-neck rotation and hyperextension caused a significant decrease of more than 20% in MCA mean BFlow compared to baseline. They stated that transcranial DUS might be valuable for preanesthetic evaluation [22].

The self-controlled design of our study, conducted in non-anesthetized healthy adults, allowed us to minimize the confounding effects of anesthesia and surgical stress, although direct statistical interaction analyses were not possible. Based on our findings, we conclude that the underlying mechanism in maintaining brain saturation is the maintenance of cerebral arterial blood flow via the vertebrobasilar system, as we did not observe any changes in vertebral artery hemodynamic measurements. Moreover, the fact that signs of acute cerebral congestion occurred in only 30 min, even in healthy volunteers who were not under the impact of anesthesia, emphasizes the importance of close monitoring techniques during prolonged operations and even the search for positions more innocuous than cervical hyperextension. The significant decrease in SO values after cervical hyperextension and maintenance in each period in our study is attributed to our volunteers being followed up with room air, and no additional oxygen was applied. Although a statistically significant decrease was observed, the SO values of all volunteers at each period were within the normal physiological limits.

Our study has some limitations. Most importantly, the cognitive examinations were administered at T0, which caused a learning bias and increased the scores obtained when repeated only 30 min later. Another factor is that the announcement was made to the personnel in the study center while collecting the sample group. As a result, their level of education was higher than that of the general public. Therefore, it is difficult to generalize the cognitive findings to the general population. Another limitation is that the study was planned for 30 min based on previous studies regarding hemodynamic changes and our projections of discomfort caused by cervical hyperextension in awake individuals. While the self-controlled design effectively minimized potential confounding variables, the study’s further limitations include the inability to conduct direct comparisons with anesthetized or surgical populations and the lack of a parallel control group, which restricts the capacity to draw causal inferences. Consequently, our findings should be interpreted with caution, and future research incorporating appropriate control groups is necessary. Given that our study involved non-anesthetized volunteers, we strictly adhered to ethical guidelines, as well as the sample size determined by the power analysis and 30 min test duration. However, future studies with larger sample sizes and extended experimental durations are required to enhance the generalizability of our findings.

## 5. Conclusions

Our findings suggest that cervical hyperextension may contribute to acute cerebral congestion by impairing jugular venous drainage. A significant change in ONSD indirectly precedes an increase in intracranial pressure caused by this condition. This may be regarded as a response of cerebral autoregulation to cervical hyperextension in healthy adults, which targets the maintenance of cerebral arterial blood flow. However, acute cerebral congestion occurs in only 30 min, even in healthy adults not under general anesthesia, emphasizing the importance of monitoring techniques to be performed in prolonged operations and the importance of searching for more harmless positions than cervical hyperextension. Our results might influence clinical practice and future research by demonstrating that cervical hyperextension is an extreme position that has a negative impact on cerebral hemodynamics. We propose further studies involving longer durations of cervical hyperextension or patient groups with specific comorbidities under various surgical and anesthetic conditions.

## Figures and Tables

**Table 1:** Demographics, clinical characteristics, and cognitive function examinations.

Individuals Enrolled (n = 51)						
Age at enrollment; years				33.73 ± 8.20 (21–49)		
Sex		Male Female		28 (%54.9) 23 (%45.1)		
Educational status		High school Associate degree University graduate Postgraduate		9 (%17.6) 9 (%17.6) 8 (%15.7) 25 (%49.0)		
The angle of hyperextension; °				45.02 ± 5.09 (30–52)		
**Cognitive function examinations**	**T0**		**T30**		**d/t/p**	
MMSE	27.80 ± 2.33		28.43 ± 2.03		2.172/2.063/0.044	
MoCA	24.92 ± 3.22		26.65 ± 3.05		1.779/6.928/<0.001	

Values are presented as mean ± SD (range); number of volunteers (%). T0: Time frame before hyperextension when the basal measurements were made; T30: 30th minute after hyperextension. MMSE: Mini-Mental State Examination; MoCA: Montreal Cognitive Assessment. d: Cohen’s d effect size, t: paired samples *t* test.

**Table 2:** Doppler ultrasonographic assessment of cervical vessels at T0, T1, and T30.

	T0	T1	T30	η^2^/F/p	p_1_	p_2_	p_3_
Right CCA Diam	6.635 ± 0.691	6.516 ± 0.697	6.476 ± 0.639	0.108/6.073/0.003	**0.019**	**0.002**	0.374
Left CCA Diam	6.645 ± 0.744	6.463 ± 0.734	6.559 ± 0.679	0.094/5.198/0.007	**0.003**	0.129	0.083
Right CCA PSV	97.38 ± 22.49	110.20 ± 35.27	105.82 ± 27.78	0.152/8.952/<0.001	**0.001**	**0.005**	0.113
Left CCA PSV	106.79 ± 25.02	111.09 ± 24.45	113.21 ± 27.34	0.056/2.974/0.056	0.149	**0.024**	0.365
Right CCA ED	22.71 ± 5.38	26.04 ± 7.75	25.23 ± 6.39	0.150/8.851/<0.001	**0.001**	**0.004**	0.262
Left CCA ED	27.15 ± 6.10	29.99 ± 5.77	30.38 ± 6.54	0.201/12.569/<0.001	**<0.001**	**<0.001**	0.522
Right CCA RI	0.761 ± 0.058	0.753 ± 0.060	0.758 ± 0.067	0.011/0.551/0.578	0.227	0.746	0.513
Left CCA RI	0.738 ± 0.058	0.727 ± 0.052	0.725 ± 0.053	0.052/2.744/0.069	0.065	0.064	0.812
Right CCA BFlow	374.33 ± 89.98	392.71 ± 116.01	409.67 ± 106.43	0.044/2.301/0.105	0.308	**0.038**	0.262
Left CCA BFlow	420.67 ± 101.11	450.65 ± 109.93	456.69 ± 101.47	0.065/3.490/0.034	**0.036**	**0.014**	0.701
Right ICA Diam	5.173 ± 0.704	5.004 ± 0.627	5.000 ± 0.638	0.087/4.775/0.013	**0.019**	**0.015**	0.940
Left ICA Diam	5.057 ± 0.573	4.998 ± 0.581	4.975 ± 0.528	0.027/1.408/0.249	0.292	0.114	0.600
Right ICA PSV	84.57 ± 21.26	78.90 ± 21.35	76.82 ± 20.19	0.088/4.830/0.010	**0.031**	**0.005**	0.422
Left ICA PSV	86.43 ± 21.06	79.96 ± 22.76	79.22 ± 17.52	0.097/5.385/0.011	**0.035**	**0.002**	0.714
Right ICA ED	30.79 ± 7.28	29.93 ± 6.84	29.41 ± 6.75	0.022/1.108/0.329	0.281	0.203	0.578
Left ICA ED	32.22 ± 7.90	32.49 ± 7.68	32.47 ± 7.77	0.001/0.044/0.957	0.801	0.805	0.984
Right ICA RI	0.628 ± 0.072	0.609 ± 0.075	0.604 ± 0.081	0.044/2.294/0.115	0.140	0.078	0.580
Left ICA RI	0.609 ± 0.090	0.583 ± 0.066	0.586 ± 0.069	0.046/2.434/0.100	0.084	0.082	0.811
Right ICA BFlow	284.84 ± 77.95	274.39 ± 74.27	270.24 ± 81.01	0.017/0.889/0.414	0.304	0.208	0.736
Left ICA BFlow	300.78 ± 86.11	289.71 ± 74.33	305.47 ± 81.45	0.021/1.071/0.346	0.325	0.670	0.162
Right VJI Diam	6.573 ± 1.701	6.710 ± 1.974	6.759 ± 2.095	0.006/0.303/0.654	0.638	0.517	0.726
Left VJI Diam	6.520 ± 1.554	6.131 ± 1.579	6.251 ± 1.495	0.030/1.572/0.218	0.151	0.293	0.350
Right VJI PSV	34.49 ± 13.85	20.61 ± 11.14	20.01 ± 11.66	0.387/31.853/<0.001	**<0.001**	**<0.001**	0.664
Left VJI PSV	30.37 ± 11.72	17.51 ± 8.46	18.80 ± 11.37	0.439/39.103/<0.001	**<0.001**	**<0.001**	0.251
Right VA Diam	3.565 ± 0.608	3.604 ± 0.618	3.625 ± 0.632	0.027/1.375/0.257	0.316	0.153	0.473
Left VA Diam	3.665 ± 0.546	3.739 ± 0.577	3.710 ± 0.592	0.038/2.001/0.148	0.085	0.263	0.313
Right VA PSV	48.34 ± 14.67	50.68 ± 15.98	49.67 ± 16.05	0.016/0.815/0.427	0.262	0.509	0.466
Left VA PSV	54.69 ± 15.63	53.44 ± 16.62	50.29 ± 15.22	0.055/2.826/0.064	0.467	**0.040**	0.106
Right VA ED	15.46 ± 4.33	15.98 ± 5.17	15.90 ± 5.02	0.009/0.460/0.599	0.422	0.500	0.847
Left VA ED	17.25 ± 5.38	17.36 ± 4.94	17.21 ± 5.85	0.001/0.022/0.978	0.881	0.965	0.817
Right VA RI	0.676 ± 0.072	0.675 ± 0.071	0.674 ± 0.086	0.001/0.011/0.972	0.896	0.902	0.957
Left VA RI	0.666 ± 0.068	0.666 ± 0.062	0.659 ± 0.078	0.009/0.425/0.655	0.980	0.434	0.406
Right VA BFlow	84.20 ± 39.80	83.82 ± 35.66	88.39 ± 42.89	0.019/0.981/0.378	0.923	0.265	0.172
Left VA BFlow	99.49 ± 44.83	97.80 ± 44.51	96.18 ± 50.22	0.005/0.266/0.767	0.710	0.465	0.726

Values are presented as mean ± SD. p-values reaching statistical significance are presented in bold. p_1_: T1 vs. T0, p_2_: T30 vs. T0, p_3_: T30 vs. T1. T0: Time frame before hyperextension when the basal measurements were made; T1: First minute after hyperextension; T30: 30th minute after hyperextension. CCA: arteria carotis communis; ICA: arteria carotis interna; VJI: vena jugularis interna; VA: arteria vertebralis ||Diam: diameter; PSV: peak systolic velocity; ED: end-diastolic velocity; RI: resistive index; BFlow: blood flow volume. η^2^: partial eta squared, F: repeated measures ANOVA.

**Table 3:** Cerebral oximetric parameters: cerebral oxygen saturation and relative total, oxyhemoglobin, and deoxyhemoglobin changes.

	T0	T1	T5	T10	T15	T20	T25	T30
Right SO	69.76 ± 6.60	67.14 ± 5.37	67.45 ± 5.94	67.39 ± 5.94	67.24 ± 5.93	67.22 ± 5.70	66.90 ± 6.21	67.22 ± 6.10
η^2^/F/p	0.214/13.611/<0.001							
p_1_	-	**<0.001**	**0.001**	**<0.001**	**<0.001**	**<0.001**	**<0.001**	**<0.001**
p_2_	-	**<0.001**	0.197	0.799	0.370	0.908	0.245	0.153
Left SO	70.10 ± 6.31	67.39 ± 6.30	67.59 ± 6.55	67.37 ± 6.59	67.37 ± 6.67	67.24 ± 6.13	67.02 ± 6.32	67.14 ± 6.41
η^2^/F/p	0.307/22.200/<0.001							
p_1_	-	**<0.001**	**<0.001**	**<0.001**	**<0.001**	**<0.001**	**<0.001**	**<0.001**
p_2_	-	**<0.001**	0.370	0.330	0.999	0.563	0.326	0.589
Right ΔcHbi	0.537 ± 2.262	5.133 ± 9.733	8.002 ± 11.383	8.446 ± 12.390	7.576 ± 12.044	7.126 ± 11.882	7.241 ± 11.897	7.120 ± 11.746
η^2^/F/p	0.231/13.481/<0.001							
p_1_	-	**0.001**	**<0.001**	**<0.001**	**<0.001**	**<0.001**	**<0.001**	**<0.001**
p_2_	-	**0.001**	**<0.001**	0.372	**0.004**	0.250	0.766	0.765
Left ΔcHbi	0.374 ± 1.103	4.943 ± 8.202	7.730 ± 9.083	8.176 ± 10.476	7.339 ± 10.776	6.572 ± 10.640	6.409 ± 10.780	5.609 ± 10.851
η^2^/F/p	0.246/14.655/<0.001							
p_1_	-	**<0.001**	**<0.001**	**<0.001**	**<0.001**	**<0.001**	**<0.001**	**0.002**
p_2_	-	**<0.001**	**<0.001**	0.363	0.055	0.056	0.551	0.195
Right ΔHHbi	−0.061 ± 0.924	4.046 ± 3.863	4.450 ± 4.355	4.863 ± 5.627	4.730 ± 5.563	4.554 ± 5.703	4.900 ± 5.846	5.117 ± 5.983
η^2^/F/p	0.331/22.305/<0.001							
p_1_	-	**<0.001**	**<0.001**	**<0.001**	**<0.001**	**<0.001**	**<0.001**	**<0.001**
p_2_	-	**<0.001**	0.246	0.200	0.302	0.326	0.110	0.149
Left ΔHHbi	0.000 ± 0.687	4.037 ± 3.457	3.791 ± 3.576	4.411 ± 5.190	4.261 ± 5.236	4.246 ± 5.312	4.257 ± 5.285	4.235 ± 5.699
η^2^/F/p	0.305/19.748/<0.001							
p_1_	-	**<0.001**	**<0.001**	**<0.001**	**<0.001**	**<0.001**	**<0.001**	**<0.001**
p_2_	-	**<0.001**	0.111	0.147	0.440	0.920	0.922	0.904
Right ΔO_2_Hbi	0.570 ± 1.906	1.422 ± 6.961	3.533 ± 7.953	3.385 ± 8.211	2.922 ± 7.979	2.404 ± 7.533	2.328 ± 7.509	1.880 ± 7.333
η^2^/F/p	0.101/5.052/0.006							
p_1_	-	0.354	**0.007**	**0.013**	**0.032**	0.075	0.087	0.185
p_2_	-	0.354	**<0.001**	0.624	0.075	0.078	0.779	0.183
Left ΔO_2_Hbi	0.441 ± 1.215	0.946 ± 6.237	3.798 ± 7.042	3.522 ± 7.261	2.950 ± 7.586	2.302 ± 6.983	2.191 ± 7.375	1.263 ± 7.108
η^2^/F/p	0.135/7.039/<0.001							
p_1_	-	0.568	**0.001**	**0.004**	**0.023**	0.063	0.097	0.420
p_2_	-	0.568	**<0.001**	0.339	**0.043**	**0.032**	0.666	0.062

**Table 4:** Optic nerve sheath diameter (ONSD) measurements at T0 and T30.

	T0	T30	d/t/p
Right ONSD	6.029 ± 1.012	7.020 ± 0.837	0.769/9.196/<0.001
Left ONSD	6.151 ± 0.836	7.020 ± 0.664	0.630/9.840/<0.001

Values are presented as mean ± SD. T0: Time frame before hyperextension when basal measurements were made; T30: 30th minute after hyperextension. ONSD: Optic nerve sheath diameter. d: Cohen’s d effect size, t: paired samples *t* test.

## Data Availability

The de-identified data we analyzed are not publicly available, but requests to the corresponding author for the data will be considered on a case-by-case basis.

## Supplementary Materials

The following supporting information can be downloaded at: https://www.mdpi.com/article/10.3390/medicina61101791/s1, Supplementary Table S1: Variables measured in time frames T0, T1, T30, and between T1 and T30; Supplementary Figure S1: Comparison of peak systolic velocity measurements of right and left vena jugularis internae. The diagram shows a significant decrease immediately after the cervical hyperextension position (T1) and is maintained to the end of the position (T30); Supplementary Figure S2: Comparison of right and left cerebral oxygen saturation measurements. The diagram shows a significant decrease immediately after the cervical hyperextension position (t1) and is maintained in this manner; Supplementary Figure S3: Comparison of right and left relative changes in total (ΔcHbi), deoxygenated (ΔHHbi), and oxygenated hemoglobin (ΔO_2_Hbi). The diagram shows a significant increase in ΔcHbi and ΔHHbi immediately after the cervical hyperextension position and maintaining in this manner while ΔO_2_Hbi shows a significant increase until 15 min and maintains then near baseline values. This clinically signs for acute cerebral congestion; Supplementary Figure S4: Comparison of right and left relative changes in total (ΔcHbi), deoxygenated (ΔHHbi), and oxygenated hemoglobin (ΔO_2_Hbi). The diagram shows a significant increase in ΔcHbi and ΔHHbi immediately after the cervical hyperextension position and maintaining in this manner while ΔO_2_Hbi shows a significant increase until 15 min and maintains then near baseline values. This clinically signs for acute cerebral congestion; Supplementary Figure S5: Comparison of right and left optic nerve sheath diameter measurements.

## Abbreviations

The following abbreviations are used in this manuscript:

DUS	Doppler ultrasound
MRA	Magnetic resonance angiography
SO	Cerebral oxygen saturation
Hb	Hemoglobin
ONSD	Optic nerve sheath diameter
MMSE	Mini-Mental State Examination
MoCA	Montreal Cognitive Assessment
ΔcHbi	Relative total hemoglobin change
ΔHHbi	Relative deoxygenated hemoglobin change
ΔO_2_Hbi	Relative oxygenated hemoglobin change
Diam	Diameter
PSV	Peak systolic velocity
ED	End-diastolic velocity
RI	Resistive index
BFlow	Blood flow volume
CCA	Arteria carotis communis
ICA	Arteria carotis interna
VA	Vertebral artery
VJI	Vena jugularis interna
ONS	Optic nerve sheath
ICP	Intracranial pressure
MCA	Middle cerebral artery
