# Supplementary material for: Cervical Hyperextension Causes Acute Cerebral Congestion in Non-Anesthetized Healthy Adults: An Observational Self-Controlled Design Study
Source: Medicina (Kaunas). 2025 Oct 3;61(10):1791. doi: 10.3390/medicina61101791 (PMC12566163; doi:10.3390/medicina61101791)

Supplementary Table S1: Variables measured in time frames T0, T1, T30, and between T1 and T30.

	T0	T1	Between T1 and T30	T30
Cervical hyperextension	-	+	+	+
Cognitive tests	MMSE; MoCA	-	-	MMSE; MoCA
Cerebral oximetry, O3® parameters	SO basal<br>ΔcHbi basal<br>ΔHHbi basal<br>ΔO_2Hbi basal	SO1<br>ΔcHbi1<br>ΔHHbi1<br>ΔO_2Hbi1	SO-2-3-4-5-6<br>ΔcHbi-2-3-4-5-6<br>ΔHHbi-2-3-4-5-6<br>ΔO_2Hbi-2-3-4-5-6	SO7<br>ΔcHbi7<br>ΔHHbi7<br>ΔO_2Hbi7
Doppler Ultrasound	CCA Diam basal<br>CCA PSV basal<br>CCA ED basal<br>CCA RI basal<br>CCA Bflow basal<br>ICA Diam basal<br>ICA PSV basal<br>ICA ED basal<br>ICA RI basal<br>ICA Bflow basal<br>VJI Diam basal<br>VJI PSV basal<br>VA Diam basal<br>VA PSV basal<br>VA ED basal<br>VA RI basal<br>VA Bflow basal	CCA Diam1<br>CCA PSV1<br>CCA ED1<br>CCA RI1<br>CCA Bflow1<br>ICA Diam1<br>ICA PSV1<br>ICA ED1<br>ICA RI1<br>ICA Bflow1<br>VJI Diam1<br>VJI PSV1<br>VA Diam1<br>VA PSV1<br>VA ED1<br>VA RI1<br>VA Bflow1	-	CCA Diam30<br>CCA PSV30<br>CCA ED30<br>CCA RI30<br>CCA Bflow30<br>ICA Diam30<br>ICA PSV30<br>ICA ED30<br>ICA RI30<br>ICA Bflow30<br>VJI Diam30<br>VJI PSV30<br>VA Diam30<br>VA PSV30<br>VA ED30<br>VA RI30<br>VA Bflow30
Optic Nerve Sheath Diameter	+	-	-	+

*T0: Time frame before hyperextension when the basal measurements were made; T1: First minute after hyperextension; T30: 30th minute after hyperextension; MMSE: Mini-Mental State Examination; MoCA: Montreal Cognitive Assessment; SO: cerebral oxygen saturation; ΔcHbi: relative total hemoglobin change; ΔHHbi: relative deoxyhemoglobin change; ΔO_2Hbi: relative oxyhemoglobin change; CCA: arteria carotis communis; ICA: arteria carotis interna; VJI: vena jugularis interna; VA: arteria vertebralis; Diam: diameter; PSV: peak systolic velocity; ED: end-diastolic velocity; RI: resistive index; Bflow: blood flow volume

Figure Legends:

Supplementary Figure S1. Comparison of peak systolic velocity measurements of right and left vena jugularis internae. The diagram shows a significant decrease immediately after the cervical hyperextension position (T1) and is maintained to the end of the position (T30).

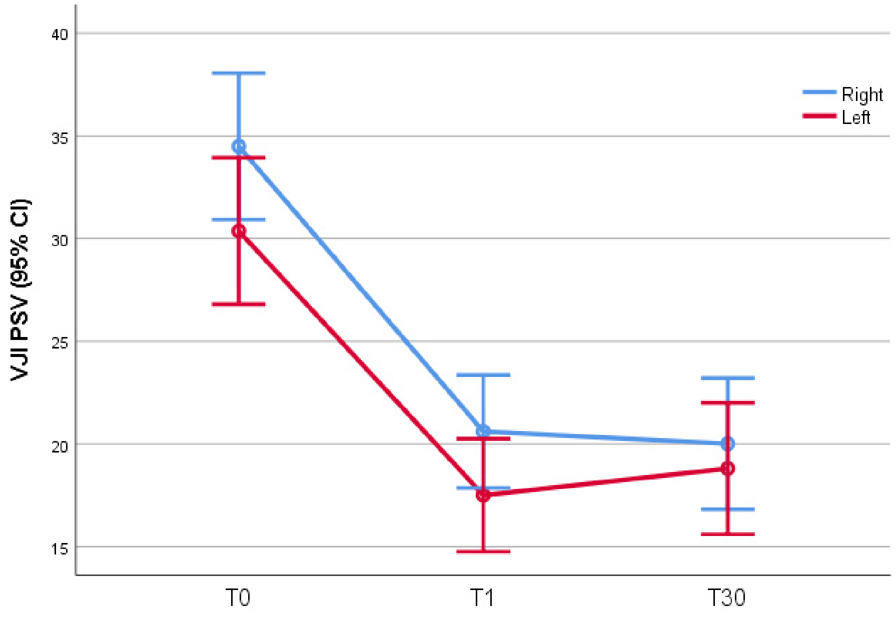

Supplementary Figure S2. Comparison of right and left cerebral oxygen saturation measurements. The diagram shows a significant decrease immediately after the cervical hyperextension position (t1) and is maintained in this manner.

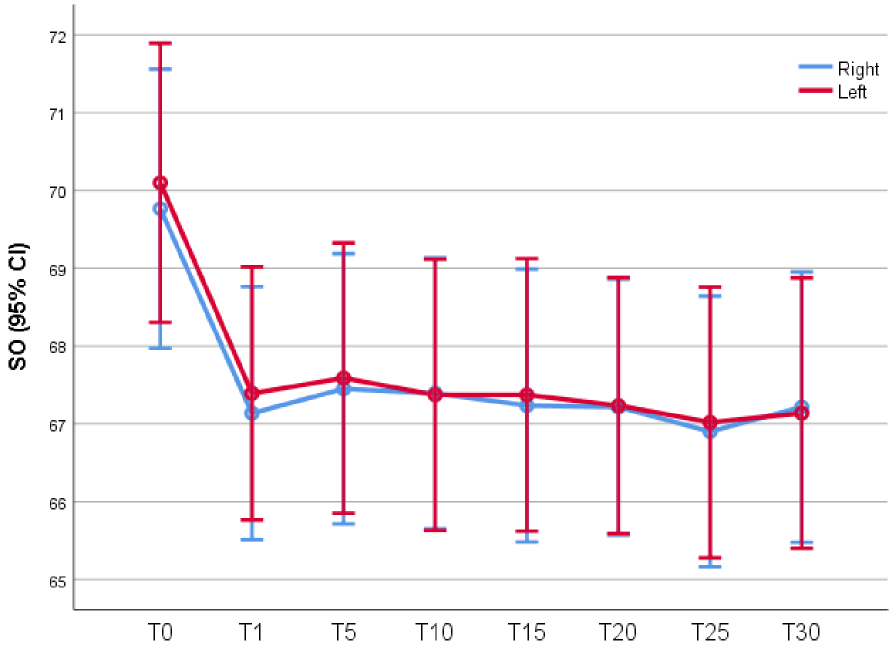

Supplementary Figure S3. Comparison of right and left relative changes in total (ΔcHbi), deoxygenated (ΔHHbi), and oxygenated hemoglobin (ΔO2Hbi). The diagram shows a significant increase in ΔcHbi and ΔHHbi immediately after the cervical hyperextension position and maintaining in this manner while ΔO2Hbi shows a significant increase until 15 minutes and maintains then near baseline values. This clinically signs for acute cerebral congestion.

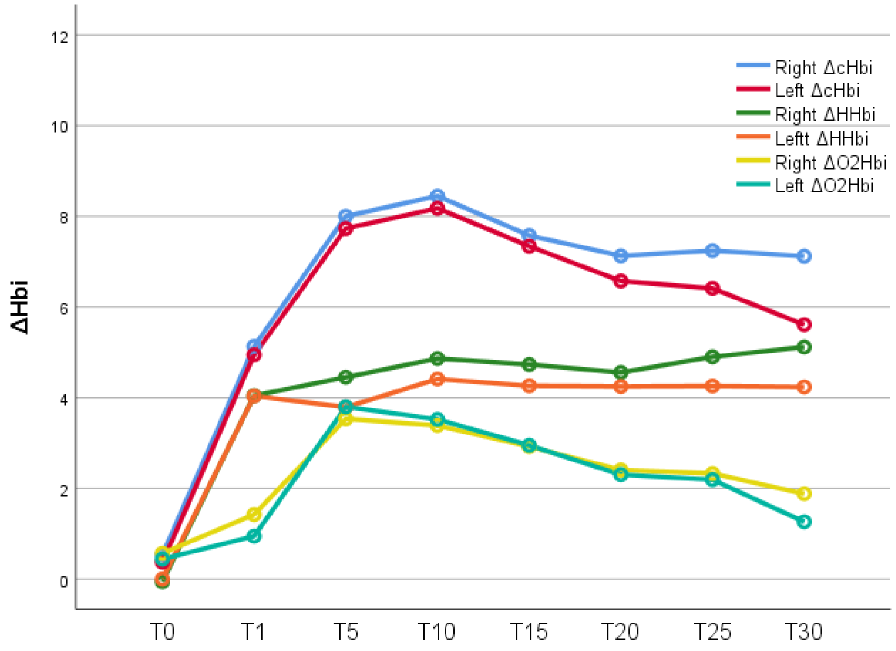

Supplementary Figure S4. Comparison of right and left relative changes in total (ΔcHbi), deoxygenated (ΔHHbi), and oxygenated hemoglobin (ΔO_2Hbi). The diagram shows a significant increase in ΔcHbi and ΔHHbi immediately after the cervical hyperextension position and maintaining in this manner while ΔO_2Hbi shows a significant increase until 15 minutes and maintains then near baseline values. This clinically signs for acute cerebral congestion.

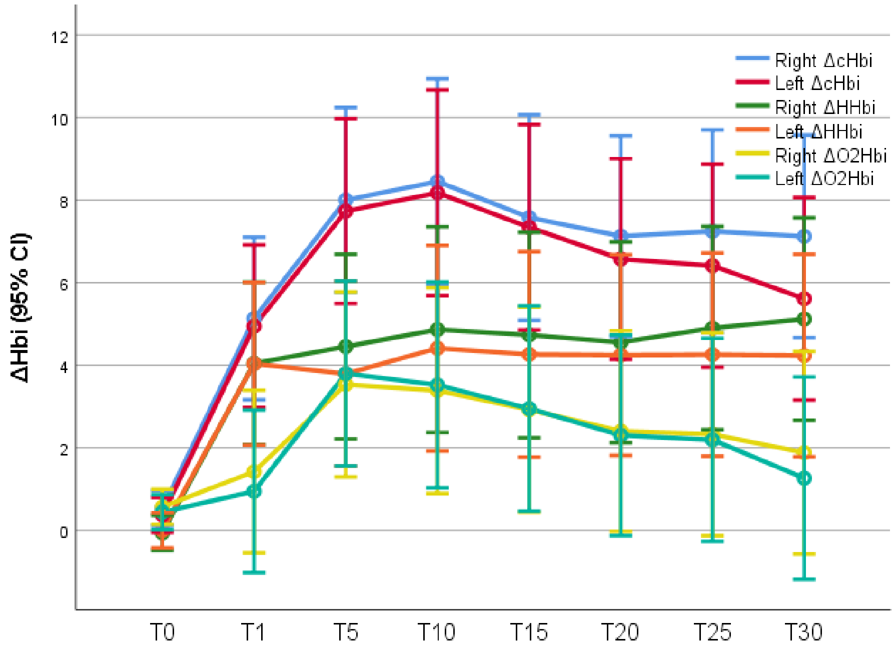

Supplementary Figure S5. Comparison of right and left optic nerve sheath diameter measurements.

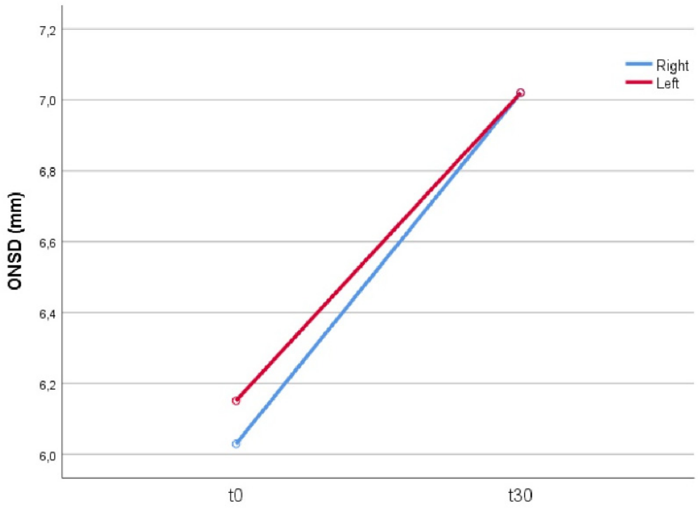

Supplement: Supplementary file 1 [file medicina-61-01791-s001.zip › 2- medicina supplementary material.pdf]
