# Supplementary figures and images for: Cervical Hyperextension Causes Acute Cerebral Congestion in Non-Anesthetized Healthy Adults: An Observational Self-Controlled Design Study
Source: Medicina (Kaunas). 2025 Oct 3;61(10):1791. doi: 10.3390/medicina61101791 (PMC12566163; doi:10.3390/medicina61101791)

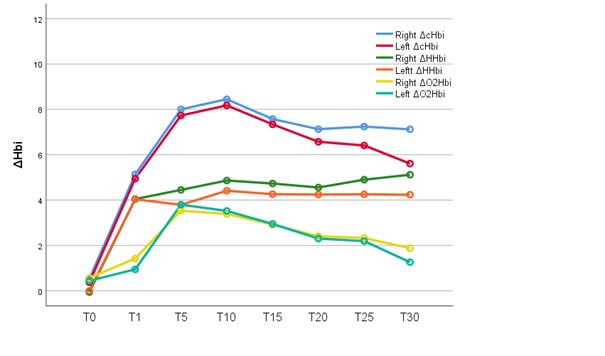

Supplement: Supplementary file 1 [file medicina-61-01791-s001.zip › 10-Supplementary Figure S3.jpg]

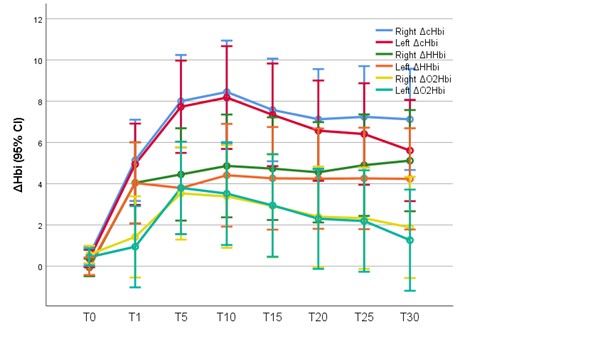

Supplement: Supplementary file 1 [file medicina-61-01791-s001.zip › 11-Supplementary Figure S4.jpg]

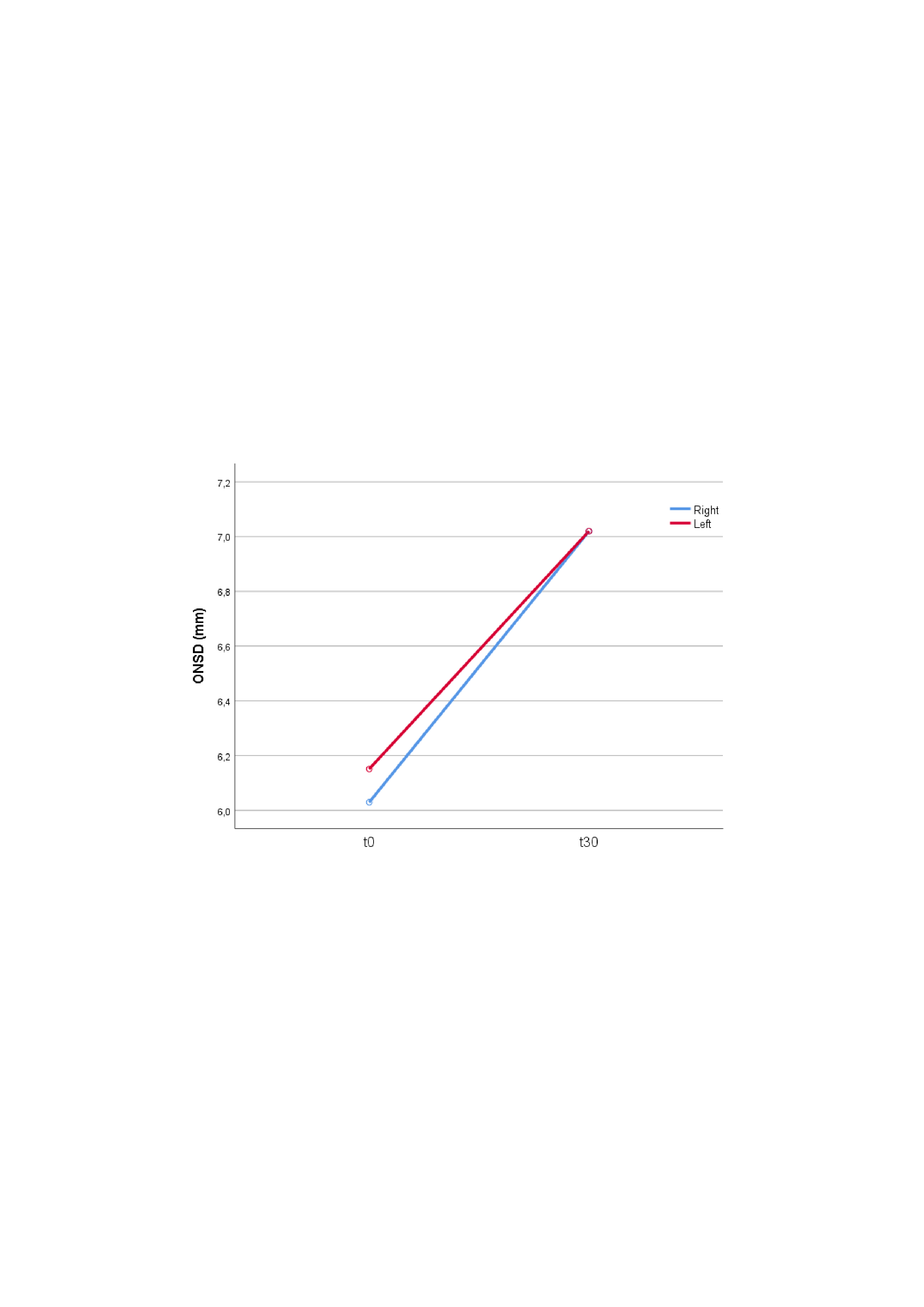

Supplement: Supplementary file 1 [file medicina-61-01791-s001.zip › 12-Supplementary Figure S5. Comparison of right and left ONSD measurements..jpg]

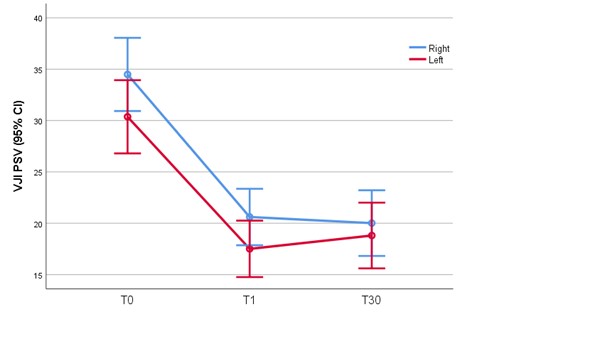

Supplement: Supplementary file 1 [file medicina-61-01791-s001.zip › 8-Supplementary Figure S1.jpg]

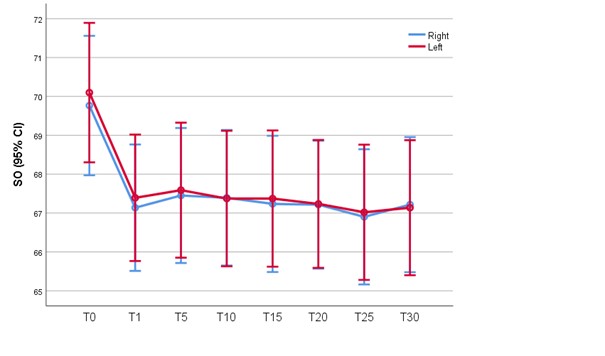

Supplement: Supplementary file 1 [file medicina-61-01791-s001.zip › 9-Supplementary Figure S2.jpg]
